# Implementation in the Motek Caren system with virtual reality of an existing motor rehabilitation programme for people with Down’s syndrome in order to increase its effectiveness

**DOI:** 10.1192/j.eurpsy.2024.1226

**Published:** 2024-08-27

**Authors:** M. A. Ciołek, K. Kamińska, A. Szczegielniak, K. Krysta, M. Krzystanek

**Affiliations:** ^1^Medical University of Silesia, Katowice, Poland; ^2^Psychiatric Rehabilitation, Medical University of Silesia, Katowice, Poland

## Abstract

**Introduction:**

Down’s syndrome often requires specialized rehabilitation methods in order to effectively improve cognitive and motor functioning. The growing interest in technologies to support rehabilitation is opening up new and promising perspectives for improving the quality of life of people diagnosed with this syndrome. One of these technologies is the Computer Assisted Rehabilitation Environment (CAREN) system from Motek.

**Objectives:**

The aim of the planned research project is to explore the potential of using the CAREN system in the rehabilitation of people with Down’s syndrome.

**Methods:**

The study included 10 participants with Down’s syndrome (men and women aged 18 to 50 years) without the presence of organic musculoskeletal disease or other somatic causes impairing motor performance. Before the training test, the participants were assessed by two psychological tests: 1) ACE III - Addenbrooke’s Cognitive Examination III Scale (ACE-III), which assesses attention and orientation, memory, verbal fluency, language and visuospatial functions and 2) the TONI 4 Non-Verbal Intelligence Test, which is a test used to measure general intelligence. The tests were carried out using the MOTEK CAREN device, which consists of a treadmill for motor training and a virtual reality screen on which different scenes are displayed for the participant to see during the test. Integrated motion capture technology was used to assess movement capabilities of the patients.. The screen displayed different types of applications in the form of virtual reality, in which the participant had to cope with various tasks accommodating different psychomotor skills, for example:crossing a virtual bridge, walking through a forest. The test took about 45 minutes per person. Two training sessions were conducted for each of the 10 patients with a one-month interval between them.

**Results:**

The Motek Caren System has proven to be a promising rehabilitation method for people with Down’s syndrome, compared to previous experience with different rehabilitation methods and existing research in the field.

**Conclusions:**

Results emphasize the necessity for further investigations and future research should involve more participants. The project has the potential to integrate modern technology with traditional forms of therapy to improve the quality of life and functioning of people affected by this syndrome.

**Disclosure of Interest:**

None Declared

